# (*E*)-3-(2,4-Dichloro­phen­yl)-1-(2-thien­yl)prop-2-en-1-one

**DOI:** 10.1107/S1600536808026524

**Published:** 2008-08-23

**Authors:** Hoong-Kun Fun, P. S. Patil, S. M. Dharmaprakash, Suchada Chantrapromma, Ibrahim Abdul Razak

**Affiliations:** a X-ray Crystallography Unit, School of Physics, Universiti Sains Malaysia, 11800 USM, Penang, Malaysia; b Department of Physics, KLE Society’s KLE Institute of Technology, Gokul Road, Hubli 590 030, India; c Department of Studies in Physics, Mangalore University, Mangalagangotri, Mangalore 574 199, India; d Crystal Materials Research Unit, Department of Chemistry, Faculty of Science, Prince of Songkla University, Hat-Yai, Songkhla 90112, Thailand

## Abstract

In the title chalcone derivative, C_13_H_8_Cl_2_OS, the prop-2-en-1-one unit and the thio­phene and 2,4-dichloro­phenyl rings are each essentially planar. The inter­planar angle between the thio­phene and 2,4-dichloro­phenyl rings is 19.87 (6)°. Weak intra­molecular C—H⋯O and C—H⋯Cl inter­actions involving the prop-2-en-1-one unit generate an *S*(5)*S*(5) ring motif. In the crystal structure, mol­ecules are linked into head-to-tail zigzag chains along the *a* axis and adjacent chains are cross-linked. These cross-linked chains are arranged into sheets parallel to the *ab* plane. The crystal structure is stabilized by weak C—H⋯O, C—H⋯Cl and C—H⋯π inter­actions. A π–π inter­action was also observed with a centroid–centroid distance of 3.6845 (6) Å.

## Related literature

For details of hydrogen-bond motifs, see: Bernstein *et al.* (1995 ▶). For bond-length data, see: Allen *et al.* (1987 ▶). For related structures, see, for example: Fun *et al.* (2008*a* ▶,*b* ▶). For background on the applications of substituted chalcones, see, for example: Agrinskaya *et al.* (1999 ▶); Chopra *et al.* (2007 ▶); Goto *et al.* (1991 ▶); Gu *et al.* (2008*a* ▶,*b* ▶,*c* ▶); Patil *et al.* (2007*a* ▶,*b* ▶,*c* ▶); Sarojini *et al.* (2006 ▶); Wang *et al.* (2004 ▶).
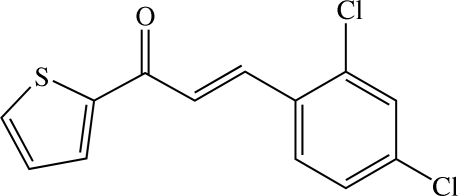


## Experimental

### 

#### Crystal data

C_13_H_8_Cl_2_OS
*M* *_r_* = 283.16
Monoclinic, 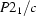

*a* = 9.5701 (4) Å
*b* = 13.9544 (6) Å
*c* = 10.4748 (4) Å
β = 118.735 (3)°
*V* = 1226.59 (9) Å^3^
*Z* = 4
Mo *K*α radiation
μ = 0.68 mm^−1^
*T* = 100.0 (1) K
0.58 × 0.24 × 0.13 mm

#### Data collection

Bruker SMART APEXII CCD area-detector diffractometer
Absorption correction: multi-scan (*SADABS*; Bruker, 2005 ▶) *T*_min_ = 0.695, *T*_max_ = 0.919
41878 measured reflections
4435 independent reflections
3831 reflections with *I* > 2σ(*I*)
*R*_int_ = 0.031

#### Refinement

*R*[*F* ^2^ > 2σ(*F* ^2^)] = 0.029
*wR*(*F* ^2^) = 0.082
*S* = 1.06
4435 reflections
162 parameters
H-atom parameters constrained
Δρ_max_ = 0.60 e Å^−3^
Δρ_min_ = −0.28 e Å^−3^

### 

Data collection: *APEX2* (Bruker, 2005 ▶); cell refinement: *APEX2*; data reduction: *SAINT* (Bruker, 2005 ▶); program(s) used to solve structure: *SHELXTL* (Sheldrick, 2008 ▶); program(s) used to refine structure: *SHELXTL*; molecular graphics: *SHELXTL*; software used to prepare material for publication: *SHELXTL* and *PLATON* (Spek, 2003 ▶).

## Supplementary Material

Crystal structure: contains datablocks global, I. DOI: 10.1107/S1600536808026524/ww2127sup1.cif

Structure factors: contains datablocks I. DOI: 10.1107/S1600536808026524/ww2127Isup2.hkl

Additional supplementary materials:  crystallographic information; 3D view; checkCIF report

## Figures and Tables

**Table 1:** Hydrogen-bond geometry (Å, °)

*D*—H⋯*A*	*D*—H	H⋯*A*	*D*⋯*A*	*D*—H⋯*A*
C3—H3*A*⋯O1^i^	0.93	2.52	3.4512 (17)	175
C7—H7*A*⋯Cl1	0.93	2.68	3.0573 (11)	105
C7—H7*A*⋯O1	0.93	2.48	2.8116 (17)	101
C10—H10*A*⋯*Cg*1^ii^	0.93	3.33	3.8233 (13)	115
C12—H12*A*⋯*Cg*1^iii^	0.93	2.87	3.6907 (13)	148

Symmetry codes: (i) 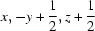; (ii) 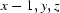; (iii) 
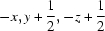
. *Cg*1 is the centroid of the S1/C1–C4 ring.

**Fig. 1.**
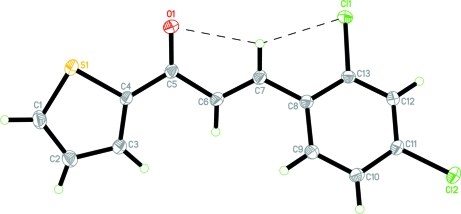
The asymmetric unit of (I), showing 50% probability displacement ellipsoids and the atomic numbering. Weak intramolecular C—H···O and C—H···Cl interactions are drawn as dashed lines.

**Fig. 2.**
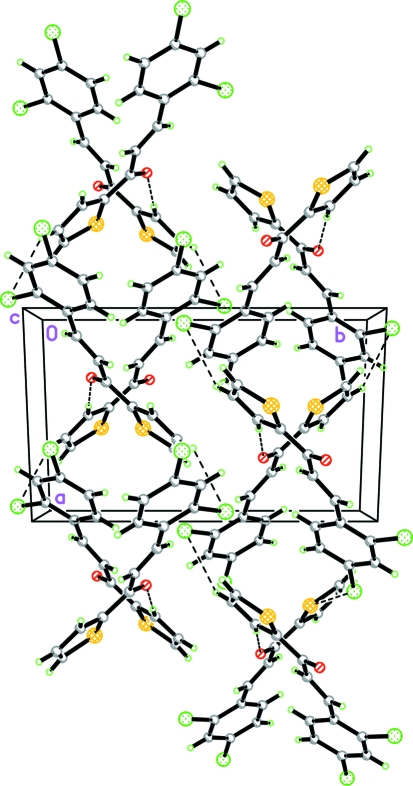
The crystal packing of (I), viewed along the c axis showing the cross-linked chains approximately along the a axis. Hydrogen bonds are drawn as dashed lines.

## supplementary crystallographic information

### Comment

In the last decades, the second-order nonlinear optical properties of chalcone derivatives have been widely investigated due to their possible applications in a variety of optoelectronic and photonic applications (Agrinskaya *et al.*, 1999; Goto *et al.*, 1991; Patil *et al.*, 2007*a*, *b*, *c*; Sarojini *et al.*, 2006; Wang *et al.*, 2004). These derivatives also exhibit the optical limiting property which is a requirement of protecting the human eye or artificial optical sensor from damaging high-energy lasers (Gu *et al.*, 2008*a*, *b*, *c*). In our continuing systematic study on chalcone derivatives, we report here the structure of the title compound.

In the structure of the title chalcone derivative (Fig. 1), bond lengths and angles are in normal ranges (Allen *et al.*, 1987) and comparable to those in related structures (Fun *et al.*, 2008*a*, *b*). The prop-2-en-1-one unit (O1/C5–C7), the thiophene ring and the 2,4-dichlorophenyl ring are individually essentially planar, with maximum deviations of 0.003 (1), 0.024 (1), -0.007 (1)Å for atom C4, C7 and C11, respectively. The total molecule is slightly twisted as indicated by the dihedral angles between the least-squares plane through the prop-2-en-1-one unit with the thiophene and 2,4-dichlorophenyl rings being 7.89 (7)° and 22.45 (7)°, and that between the thiophene and 2,4-dichlorophenyl rings being 19.87 (6)°.

In the structure, both weak intramolecular C7—H7A···O1 and C7—H7A···Cl1 interactions (Table 1) generate S(5) ring motifs (Bernstein *et al.*, 1995). In the crystal structure (Fig. 2) the molecules are linked in head-to-tail zigzag chains along the *a*-axis by weak C—H···Cl interactions and the adjacent chains were cross-linked by weak C—H···O interactions. These cross-linked chains are arranged into sheets parallel to the *ab* plane. The crystal is stabilized by weak C—H···O, C—H···Cl and C—H···π interactions (Table 1), π···π interaction was also observed with the *Cg*_2_···*Cg*_2_ distance of 3.6845 (6)Å (symmetry code: -*x*,1 - *y*, 1 - *z*); *Cg*_1_ and *Cg*_2_ are the centroids of S1/C1–C4 and C8–C13 rings.

### Experimental

The title compound was synthesized by the condensation of 2,4-dichlorobenzaldehyde (0.01 mol, 1.75 g) with 2-acetylthiophene (0.01 mol, 1.07 ml) in methanol (60 ml) in the presence of a catalytic amount of sodium hydroxide solution (5 ml, 30%). After stirring (6 h), the contents of the flask were poured into ice-cold water (500 ml) and left to stand for 5 h. The resulting crude solid was filtered and dried. Needle colorless single crystals of the title compound suitable for *X*-Ray structure determination were grown by slow evaporation of the methanol solution at room temperature.

### Refinement

All H atoms were placed in calculated positions with d(C—H) = 0.93Å, *U*_iso_=1.2*U*_eq_(C) for vinylic and aromatic H atoms. The highest residual electron density peak is located at 0.70Å from C10 and the deepest hole is located at 0.51Å from S1.

### Crystal data

**Table:**

C_13_H_8_Cl_2_OS	*F*_000_ = 576
*M**_r_* = 283.16	*D*_x_ = 1.533 Mg m^−^^3^
Monoclinic, *P*2_1_/*c*	Mo *K*α radiation λ = 0.71073 Å
Hall symbol: -P 2ybc	Cell parameters from 4435 reflections
*a* = 9.5701 (4) Å	θ = 2.4–32.5º
*b* = 13.9544 (6) Å	µ = 0.68 mm^−^^1^
*c* = 10.4748 (4) Å	*T* = 100.0 (1) K
β = 118.735 (3)º	Needle, colorless
*V* = 1226.59 (9) Å^3^	0.58 × 0.24 × 0.13 mm
*Z* = 4	

### Data collection

**Table:**

Bruker SMART APEX2 CCD area-detector diffractometer	4435 independent reflections
Radiation source: fine-focus sealed tube	3831 reflections with *I* > 2σ(*I*)
Monochromator: graphite	*R*_int_ = 0.031
Detector resolution: 8.33 pixels mm^-1^	θ_max_ = 32.5º
*T* = 100.0(1) K	θ_min_ = 2.4º
ω scans	*h* = −14→14
Absorption correction: multi-scan(SADABS; Bruker, 2005)	*k* = −21→21
*T*_min_ = 0.695, *T*_max_ = 0.919	*l* = −15→15
41878 measured reflections	

### Refinement

**Table:**

Refinement on *F*^2^	Secondary atom site location: difference Fourier map
Least-squares matrix: full	Hydrogen site location: inferred from neighbouring sites
*R*[*F*^2^ > 2σ(*F*^2^)] = 0.029	H-atom parameters constrained
*wR*(*F*^2^) = 0.082	*w* = 1/[σ^2^(*F*_o_^2^) + (0.0403*P*)^2^ + 0.4876*P*] where *P* = (*F*_o_^2^ + 2*F*_c_^2^)/3
*S* = 1.07	(Δ/σ)_max_ = 0.001
4435 reflections	Δρ_max_ = 0.60 e Å^−^^3^
162 parameters	Δρ_min_ = −0.28 e Å^−^^3^
Primary atom site location: structure-invariant direct methods	Extinction correction: none

### Special details

**Table:**

Experimental. The low-temperature data was collected with the Oxford Cyrosystem Cobra low-temperature attachment.
Geometry. All e.s.d.'s (except the e.s.d. in the dihedral angle between two l.s. planes) are estimated using the full covariance matrix. The cell e.s.d.'s are taken into account individually in the estimation of e.s.d.'s in distances, angles and torsion angles; correlations between e.s.d.'s in cell parameters are only used when they are defined by crystal symmetry. An approximate (isotropic) treatment of cell e.s.d.'s is used for estimating e.s.d.'s involving l.s. planes.
Refinement. Refinement of *F*^2^ against ALL reflections. The weighted *R*-factor *wR* and goodness of fit *S* are based on *F*^2^, conventional *R*-factors *R* are based on *F*, with *F* set to zero for negative *F*^2^. The threshold expression of *F*^2^ > σ(*F*^2^) is used only for calculating *R*-factors(gt) *etc*. and is not relevant to the choice of reflections for refinement. *R*-factors based on *F*^2^ are statistically about twice as large as those based on *F*, and *R*- factors based on ALL data will be even larger.

### Fractional atomic coordinates and isotropic or equivalent isotropic displacement parameters (Å^2^)

**Table:**

	*x*	*y*	*z*	*U*_iso_*/*U*_eq_	
Cl1	−0.05796 (3)	0.558919 (18)	0.18442 (3)	0.01990 (7)	
Cl2	−0.34903 (3)	0.43921 (2)	0.48214 (3)	0.02181 (7)	
S1	0.55248 (3)	0.18039 (2)	0.20853 (3)	0.02097 (7)	
O1	0.30678 (11)	0.32994 (6)	0.13125 (9)	0.02122 (16)	
C1	0.64865 (14)	0.08866 (9)	0.32542 (13)	0.0239 (2)	
H1A	0.7245	0.0504	0.3162	0.042 (5)*	
C2	0.60409 (14)	0.07946 (9)	0.43128 (13)	0.0222 (2)	
H2A	0.6448	0.0331	0.5039	0.030 (4)*	
C3	0.48923 (13)	0.14851 (8)	0.41751 (12)	0.01776 (19)	
H3A	0.4462	0.1531	0.4802	0.025 (4)*	
C4	0.44866 (12)	0.20851 (7)	0.29930 (11)	0.01483 (17)	
C5	0.32870 (12)	0.28469 (7)	0.24030 (11)	0.01551 (18)	
C6	0.23178 (13)	0.30140 (8)	0.31373 (11)	0.01662 (18)	
H6A	0.2532	0.2670	0.3972	0.033 (4)*	
C7	0.11366 (13)	0.36566 (7)	0.26054 (12)	0.01658 (18)	
H7A	0.0995	0.4012	0.1800	0.022 (4)*	
C8	0.00490 (12)	0.38454 (7)	0.31897 (11)	0.01508 (17)	
C9	−0.01998 (13)	0.31711 (8)	0.40561 (12)	0.01814 (19)	
H9A	0.0373	0.2601	0.4292	0.032 (4)*	
C10	−0.12712 (13)	0.33288 (8)	0.45700 (12)	0.01822 (19)	
H10A	−0.1418	0.2872	0.5141	0.030 (4)*	
C11	−0.21239 (12)	0.41837 (8)	0.42150 (12)	0.01651 (18)	
C12	−0.19070 (12)	0.48797 (7)	0.33822 (11)	0.01626 (18)	
H12A	−0.2472	0.5453	0.3164	0.023 (4)*	
C13	−0.08277 (12)	0.47006 (7)	0.28828 (11)	0.01535 (18)	

### Atomic displacement parameters (Å^2^)

**Table:**

	*U*^11^	*U*^22^	*U*^33^	*U*^12^	*U*^13^	*U*^23^
Cl1	0.02152 (13)	0.01786 (12)	0.02316 (13)	0.00278 (8)	0.01301 (10)	0.00674 (9)
Cl2	0.02120 (13)	0.02533 (13)	0.02547 (14)	0.00247 (9)	0.01647 (11)	0.00112 (10)
S1	0.02269 (14)	0.02431 (14)	0.02117 (13)	0.00481 (10)	0.01474 (11)	0.00138 (10)
O1	0.0269 (4)	0.0218 (4)	0.0201 (4)	0.0047 (3)	0.0155 (3)	0.0046 (3)
C1	0.0216 (5)	0.0255 (5)	0.0248 (5)	0.0084 (4)	0.0113 (4)	0.0015 (4)
C2	0.0221 (5)	0.0233 (5)	0.0201 (5)	0.0049 (4)	0.0092 (4)	0.0019 (4)
C3	0.0195 (5)	0.0187 (4)	0.0167 (4)	0.0009 (4)	0.0100 (4)	−0.0006 (4)
C4	0.0159 (4)	0.0156 (4)	0.0155 (4)	0.0001 (3)	0.0096 (4)	−0.0013 (3)
C5	0.0176 (4)	0.0152 (4)	0.0154 (4)	−0.0004 (3)	0.0093 (4)	−0.0019 (3)
C6	0.0191 (5)	0.0178 (4)	0.0159 (4)	0.0006 (4)	0.0107 (4)	−0.0001 (3)
C7	0.0191 (4)	0.0160 (4)	0.0178 (4)	0.0001 (3)	0.0114 (4)	−0.0007 (3)
C8	0.0162 (4)	0.0147 (4)	0.0154 (4)	0.0005 (3)	0.0084 (4)	−0.0001 (3)
C9	0.0211 (5)	0.0148 (4)	0.0216 (5)	0.0023 (3)	0.0127 (4)	0.0018 (4)
C10	0.0215 (5)	0.0162 (4)	0.0208 (5)	0.0005 (4)	0.0133 (4)	0.0016 (4)
C11	0.0160 (4)	0.0187 (4)	0.0170 (4)	0.0000 (3)	0.0097 (4)	−0.0015 (3)
C12	0.0155 (4)	0.0162 (4)	0.0173 (4)	0.0018 (3)	0.0081 (4)	0.0006 (3)
C13	0.0158 (4)	0.0148 (4)	0.0154 (4)	−0.0003 (3)	0.0075 (3)	0.0016 (3)

### Geometric parameters (Å, °)

**Table:**

Cl1—C13	1.7396 (10)	C6—C7	1.3367 (15)
Cl2—C11	1.7310 (11)	C6—H6A	0.9300
S1—C1	1.7033 (12)	C7—C8	1.4629 (14)
S1—C4	1.7186 (10)	C7—H7A	0.9300
O1—C5	1.2317 (13)	C8—C13	1.4042 (14)
C1—C2	1.3720 (17)	C8—C9	1.4048 (15)
C1—H1A	0.9425	C9—C10	1.3858 (16)
C2—C3	1.4160 (16)	C9—H9A	0.9300
C2—H2A	0.9300	C10—C11	1.3912 (15)
C3—C4	1.3865 (15)	C10—H10A	0.9301
C3—H3A	0.9302	C11—C12	1.3862 (15)
C4—C5	1.4656 (14)	C12—C13	1.3866 (15)
C5—C6	1.4806 (14)	C12—H12A	0.9299
			
C1—S1—C4	91.72 (6)	C6—C7—H7A	117.4
C2—C1—S1	112.39 (9)	C8—C7—H7A	117.4
C2—C1—H1A	125.7	C13—C8—C9	116.67 (9)
S1—C1—H1A	121.9	C13—C8—C7	121.62 (9)
C1—C2—C3	112.43 (11)	C9—C8—C7	121.69 (9)
C1—C2—H2A	123.8	C10—C9—C8	122.10 (10)
C3—C2—H2A	123.8	C10—C9—H9A	119.0
C4—C3—C2	111.85 (10)	C8—C9—H9A	118.9
C4—C3—H3A	124.1	C9—C10—C11	118.74 (10)
C2—C3—H3A	124.1	C9—C10—H10A	120.6
C3—C4—C5	129.85 (9)	C11—C10—H10A	120.6
C3—C4—S1	111.60 (8)	C12—C11—C10	121.53 (10)
C5—C4—S1	118.44 (8)	C12—C11—Cl2	118.77 (8)
O1—C5—C4	120.84 (9)	C10—C11—Cl2	119.70 (8)
O1—C5—C6	122.07 (10)	C11—C12—C13	118.37 (10)
C4—C5—C6	117.04 (9)	C11—C12—H12A	120.8
C7—C6—C5	120.30 (10)	C13—C12—H12A	120.8
C7—C6—H6A	119.9	C12—C13—C8	122.57 (9)
C5—C6—H6A	119.8	C12—C13—Cl1	117.25 (8)
C6—C7—C8	125.15 (10)	C8—C13—Cl1	120.18 (8)
			
C4—S1—C1—C2	−0.23 (10)	C6—C7—C8—C9	−21.13 (17)
S1—C1—C2—C3	−0.06 (14)	C13—C8—C9—C10	0.92 (16)
C1—C2—C3—C4	0.42 (15)	C7—C8—C9—C10	−177.58 (10)
C2—C3—C4—C5	175.55 (11)	C8—C9—C10—C11	−0.07 (17)
C2—C3—C4—S1	−0.59 (12)	C9—C10—C11—C12	−0.93 (17)
C1—S1—C4—C3	0.47 (9)	C9—C10—C11—Cl2	179.10 (9)
C1—S1—C4—C5	−176.16 (9)	C10—C11—C12—C13	0.99 (16)
C3—C4—C5—O1	−178.54 (11)	Cl2—C11—C12—C13	−179.04 (8)
S1—C4—C5—O1	−2.62 (14)	C11—C12—C13—C8	−0.07 (16)
C3—C4—C5—C6	−1.06 (16)	C11—C12—C13—Cl1	−179.86 (8)
S1—C4—C5—C6	174.85 (7)	C9—C8—C13—C12	−0.86 (15)
O1—C5—C6—C7	1.53 (16)	C7—C8—C13—C12	177.65 (10)
C4—C5—C6—C7	−175.91 (10)	C9—C8—C13—Cl1	178.93 (8)
C5—C6—C7—C8	176.55 (10)	C7—C8—C13—Cl1	−2.57 (14)
C6—C7—C8—C13	160.45 (11)		

### Hydrogen-bond geometry (Å, °)

**Table:**

*D*—H···*A*	*D*—H	H···*A*	*D*···*A*	*D*—H···*A*
C3—H3A···O1^i^	0.93	2.52	3.4512 (17)	175
C7—H7A···Cl1	0.93	2.68	3.0573 (11)	105
C7—H7A···O1	0.93	2.48	2.8116 (17)	101
C10—H10A···Cg1^ii^	0.93	3.33	3.8233 (13)	115
C12—H12A···Cg1^iii^	0.93	2.87	3.6907 (13)	148

Symmetry codes: (i) *x*, −*y*+1/2, *z*+1/2;  (ii) *x*−1, *y*, *z*; (iii) −*x*, *y*+1/2, −*z*+1/2.

## References

[bb1] Agrinskaya, N. V., Lukoshkin, V. A., Kudryavtsev, V. V., Nosova, G. I., Solovskaya, N. A. & Yakimanski, A. V. (1999). *Phys. Solid State* **41**, 1914–1917.

[bb2] Allen, F. H., Kennard, O., Watson, D. G., Brammer, L., Orpen, A. G. & Taylor, R. (1987). *J. Chem. Soc. Perkin Trans. 2*, pp. S1–S19.

[bb3] Bernstein, J., Davis, R. E., Shimoni, L. & Chang, N.-L. (1995). *Angew. Chem. Int. Ed. Engl* **34**, 1555–1573.

[bb4] Bruker (2005). *APEX2*, *SAINT* and *SADABS* Bruker AXS Inc., Madison, Wisconsin, USA.

[bb5] Chopra, D., Mohan, T. P., Vishalakshi, B. & Guru Row, T. N. (2007). *Acta Cryst.* C**63**, o704–o710. 10.1107/S0108270107049426 18057618

[bb6] Fun, H.-K., Chantrapromma, S., Patil, P. S. & Dharmaprakash, S. M. (2008*b*). *Acta Cryst.* E**64**, o1720–o1721. 10.1107/S1600536808024872 PMC2960501 21201705

[bb7] Fun, H.-K., Jebas, S. R., Patil, P. S. & Dharmaprakash, S. M. (2008*a*). *Acta Cryst.* E**64**, o1510–o1511. 10.1107/S1600536808021375 PMC2962137 21203219

[bb8] Goto, Y., Hayashi, A., Kimura, Y. & Nakayama, M. (1991). *J. Cryst. Growth* **108**, 688–698.

[bb9] Gu, B., Ji, W. & Huang, X.-Q. (2008*a*). *Appl. Optics* **47**, 1187–1192. 10.1364/ao.47.001187 18709063

[bb10] Gu, B., Ji, W., Patil, P. S. & Dharmaprakash, S. M. (2008*b*). *J. Appl. Phys* **103**, 103511-1–103511-6.

[bb11] Gu, B., Ji, W., Patil, P. S., Dharmaprakash, S. M. & Wang, H. T. (2008*c*). *Appl. Phys. Lett* **92**, 091118-1–091118-3.

[bb12] Patil, P. S., Dharmaprakash, S. M., Ramakrishna, K., Fun, H.-K., Sai Santosh Kumar, R. & Rao, D. N. (2007*a*). *J. Cryst. Growth* **303**, 520–524.

[bb13] Patil, P. S., Fun, H.-K., Chantrapromma, S. & Dharmaprakash, S. M. (2007*b*). *Acta Cryst.* E**63**, o2497–o2498.

[bb14] Patil, P. S., Teh, J. B.-J., Fun, H.-K., Razak, I. A. & Dharmaprakash, S. M. (2007*c*). *Acta Cryst.* E**63**, o2122–o2123.

[bb15] Sarojini, B. K., Narayana, B., Ashalatha, B. V., Indira, J. & Lobo, K. G. (2006). *J. Cryst. Growth* **295**, 54–59.

[bb16] Sheldrick, G. M. (2008). *Acta Cryst.* A**64**, 112–122. 10.1107/S0108767307043930 18156677

[bb17] Spek, A. L. (2003). *J. Appl. Cryst.* **36**, 7–13.

[bb18] Wang, L., Zhang, Y., Lu, C.-R. & Zhang, D.-C. (2004). *Acta Cryst.* C**60**, o696–o698. 10.1107/S0108270104017883 15345860

